# Supplementary material for: Splice-Junction-Based Mapping of Alternative Isoforms in the Human Proteome
Source: Cell Rep. Author manuscript; Available in PMC 2020 Jan 15. (PMC6961840; doi:10.1016/j.celrep.2019.11.026)
Supplement: 2 [file NIHMS1546469-supplement-2.zip › DF1/PXD006675/CardiacFibroblast/CardiacFibroblast_27_TTN_VTEPPYFVEKPQSQDVNPNTR.pdf]

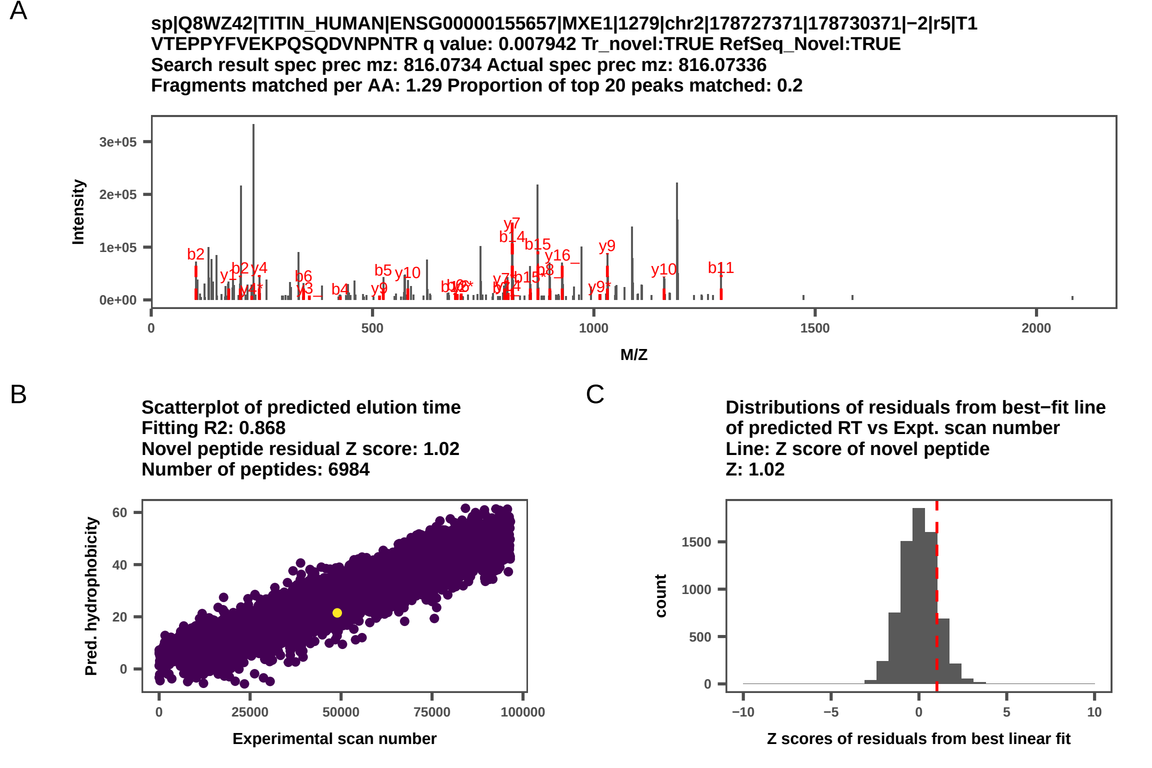
A
sp|Q8WZ42|TITIN_HUMAN|ENSG00000155657|MXE1|1279|chr2|178727371|178730371|−2|r5|T1
VTEPPYFVEKPQSQDVNPNTR q value: 0.007942 Tr_novel:TRUE RefSeq_Novel:TRUE
Search result spec prec mz: 816.0734 Actual spec prec mz: 816.07336
Fragments matched per AA: 1.29 Proportion of top 20 peaks matched: 0.2
Intensity
3e+05
2e+05
1e+05
0e+00
0
500
1000
1500
2000
M/Z
b2
y4
b6
b4
b5
y10
y7
b14
b15
y16
y9
y10
b11
B
Scatterplot of predicted elution time
Fitting R2: 0.868
Novel peptide residual Z score: 1.02
Number of peptides: 6984
Pred. hydrophobicity
60
40
20
0
0
25000
50000
75000
100000
Experimental scan number
C
Distributions of residuals from best−fit line
of predicted RT vs Expt. scan number
Line: Z score of novel peptide
Z: 1.02
count
1500
1000
500
0
−10
−5
0
5
10
Z scores of residuals from best linear fit
